# A potential oral microbiome signature associated with coronary artery disease in Tunisia

**DOI:** 10.1042/BSR20220583

**Published:** 2022-07-01

**Authors:** Fériel Bouzid, Imen Gtif, Suad Alfadhli, Salma Charfeddine, Walid Ghorbel, Rania Abdelhédi, Riadh Benmarzoug, Leila Abid, Nouha Bouayed Abdelmoula, Inés Elloumi, Saber Masmoudi, Ahmed Rebai, Najla Kharrat

**Affiliations:** 1 Laboratory of Molecular and Cellular Screening Processes, Centre of Biotechnology of Sfax, University of Sfax, Sfax, Tunisia; 2 Department of Medical Laboratory, Faculty of Allied Health, Kuwait University, Sulaibekhat 90805, State of Kuwait; 3 Department of Cardiology, Hédi Chaker University Hospital, Faculty of Medicine of Sfax, University of Sfax, Tunisia; 4 Department of Dentistry, Hédi Chaker University Hospital, Faculty of Dental Medicine of Monastir, University of Monastir, Tunisia; 5 Department of Histology: UR17ES36 Genomics of Signalopathies in the Service of Medicine, University of Sfax, Tunisia

**Keywords:** atherosclerosis, coronary artery disease, Eikenella, NGS, Oral microbiome, Tunisia

## Abstract

The coronary artery disease (CAD) is a chronic inflammatory disease involving genetic as well as environmental factors. Recent evidence suggests that the oral microbiome has a significant role in triggering atherosclerosis. The present study assessed the oral microbiome composition variation between coronary patients and healthy subjects in order to identify a potential pathogenic signature associated with CAD. We performed metagenomic profiling of salivary microbiomes by 16S ribosomal RNA (rRNA) next-generation sequencing. Oral microbiota profiling was performed for 30 individuals including 20 patients with CAD and ten healthy individuals without carotid plaques or previous stroke or myocardial infarction.

We found that oral microbial communities in patients and healthy controls are represented by similar global core oral microbiome. The predominant taxa belonged to Firmicutes (genus *Streptococcus*, *Veillonella*, *Granulicatella, Selenomonas*), Proteobacteria (genus *Neisseria*, *Haemophilus*), Actinobacteria (genus *Rothia*), Bacteroidetes (genus *Prevotella*, *Porphyromonas*), and Fusobacteria (genus *Fusobacterium, Leptotrichia*). More than 60% relative abundance of each sample for both CAD patients and controls is represented by three major genera including *Streptococcus* (24.97 and 26.33%), *Veillonella* (21.43 and 19.91%), and *Neisseria* (14.23 and 15.33%).

Using penalized regression analysis, the bacterial genus *Eikenella* was involved as the major discriminant genus for both status and Syntax score of CAD. We also reported a significant negative correlation between Syntax score and *Eikenella* abundance in coronary patients’ group (Spearman rho = −0.68, *P=0.00094*).

In conclusion, the abundance of *Eikenella* in oral coronary patient samples compared with controls could be a prominent pathological indicator for the development of CAD.

## Introduction

The oral route is the central channel for entry of bacteria into the human body. The oral cavity contains the second most complex and unique microbial communities in the human body after the gut microbiome [1]. These microorganisms colonize periodontal surfaces and are part of the saliva [1]. The dynamic and polymicrobial oral microbiome includes more than 700 commensal and pathogenic bacterial species but remains susceptible to alteration due to infections or other stress factors [2].

In recent years, intense research has highlighted an increasing significance of the oral microbiome in human health and disease [1]. A growing number of studies have shown the involvement of oral microbiome in several systemic diseases such as rheumatoid arthritis, diabetes, and cardiovascular diseases (CVD) [1].

CVD are the first cause of death globally, being responsible of 17.9 million deaths worldwide in 2019, among which 31680 occurred in Tunisia [3,4]. Notably, CVD includes coronary artery disease (CAD) that is an inflammatory disorder caused by the buildup of atherosclerotic plaques in the heart’s arteries after cholesterol accumulation and inflammation due to genetic as well as environmental factors that could lead to heart attack [5]. CAD mortality rates in Tunisia increased by 680 additional deaths between 1997 and 2009 representing an increase of 11.8% for men and 23.8% for women [6]. Recent evidence suggests that the host microbiota may be considered as an environmental factor, which plays a significant role in triggering atherosclerosis [7]. With the development of targeted microbial techniques, a number of oral bacteria have been found in atherosclerotic plaque samples from CAD patients suggesting that the oral cavity might serve as a reservoir of these potentially pathogenic microorganisms [7,8].

An established association exists between periodontal disease and CAD that was first demonstrated in the 90s by De Stefano et al., who reported a 25% increased risk of atherosclerotic plaque formation in periodontitis patients [9].

Oral bacteria and their products (toxins and/or secreted proteins) have access to the blood circulation directly through inflamed oral tissues and indirectly via saliva through the gastrointestinal tract, resulting in systemic inflammatory and immunologic responses [10]. Therefore, away from the oral cavity, atherosclerotic plaque formation may be initiated through an inflammatory stimulus induced by oral bacteria, in particular those associated with oral infectious diseases and adapted to thrive in an inflammatory environment (e.g., caries and periodontitis) [10].

To address the question if an altered oral microbiota is associated with atherosclerosis compared with healthy subjects, 16S ribosomal RNA (rRNA)-based metagenomic analysis was conducted in several studies for different study populations: in Sweden [11,12], Germany [13], and India [14]. Abundance of *Anaeroglobus* in the oral cavity was associated with symptomatic atherosclerosis [12]. However, atherosclerosis was not related to significant qualitative changes of the oral microbiota compared with healthy controls in other studies [11,13,14].

Here, we report, for the first time in Tunisia and North Africa, the characterization of the oral microbiota of patients with CAD and healthy controls by 16S rRNA-based metagenomics. This salivary microbiome profiling aims to elucidate potential qualitative and/or quantitative changes of the oral microbiota as predisposing factor and/or pathogenic signature associated with CAD.

## Materials and methods

### Study population

The present study was approved by the Local Ethics Committee of CHU Hédi Chaker of Sfax (Tunisia), according to the principles expressed in the Declaration of Helsinki and subsequent revisions (*HCH*2014-05-20). The study was conducted in collaboration with the Department of Cardiology and Department of Dentistry of Hédi Chaker University Hospital (CHU Hédi Chaker) of Sfax (Tunisia). Written informed consent was obtained from all participants prior to the study.

Twenty patients with CAD were enrolled in the study from patients of the CHU Hédi Chaker of Sfax between November 2018 and December 2019. Patients’ recruitment was done according to the following criteria: non-smoking, over age 45, free from other inflammatory diseases, no taking treatment with antibiotics or inflammatory drugs within the last 3 months and non-pregnancy at the study period (for women). Dental examination was performed to exclude individuals with dental caries susceptible to alter the oral microbiota. CAD patients were subjected to a medical examination to quantify the degree of internal carotid artery stenosis by angiography imaging and were classified according to their stenosis status in two categories: non-significant coronary artery stenosis (stenosis <50%); significant coronary artery stenosis (stenosis ≥50%). The Syntax score, an angiographic tool grading the complexity of CAD, was calculated for all patients by an experienced cardiologist. Thereafter, the Syntax score was divided into three groups: low (≤16), intermediate (16–22), and high (>22) [15].

Ten healthy subjects free from CAD and fulfilling the same inclusion criteria were included in the study. All participants answered an anonymized questionnaire containing personal information (age, sex, nationality, address, job), medical history, and nutritional habits.

To evaluate the Mediterranean diet (MD) adherence, we used an MD score proposed by Trichopoulou et al. (1995) [16]. The MD adherence score was subsequently dichotomized into two groups: 0 ≤ score < 4 represented poor adherence to MD and 4 ≤ score ≤ 8 represented strong adherence to MD.

### Samples collection

For oral microbiome profiling, saliva samples were collected from all recruited subjects in sterile tube under fasting conditions to avoid the effects of diet on the oral microbiota. Each sample was placed in an ice bag immediately and delivered to the laboratory within 2 h. DNA was extracted on fresh saliva samples upon arrival to the laboratory using QIAamp DNA Microbiome Kit (Qiagen, Hilden, Germany), following the manufacturer’s instructions and stored at −20° until analysis. Briefly, the protocol of the QIAamp DNA Microbiome Kit is designed to enzymatically deplete host DNA prior to bacterial cell lysis and to disrupt bacterial cells by a combination of mechanical and chemical lysis. In each DNA extraction series, one blank control was used to confirm the absence of contamination.

### PCR amplification and next-generation sequencing of 16S rRNA genes

Thirty oral genomic DNAs were quantified by a Qubit assay with the high sensitivity kit (Life technologies, Carlsbad, CA, U.S.A.) and then calibrated to 5 ng/μl in 10 mM Tris (pH 8.5) as recommended by Illumina guide. 16S rRNA gene amplicon libraries were prepared by PCR amplification of an approximate 460 bp within the hypervariable V3–V4 region of the 16S rRNA gene with specific primers according to the Illumina 16S metagenomic sequencing library protocol, with modifications. To test for contaminating 16S DNA during the 16S library preparation, two negative control reactions were conducted by substituting template DNA by molecular grade water and were processed at the same time and with the same reagents as samples. Cycle conditions were 95°C (45 s), then 25 cycles of 95°C (15 s), 60°C (15 s), 72°C (30 s), then a final extension of 72°C (1 min). The amplified products were checked by agarose gel electrophoresis. Libraries were purified using AMPure XP beads (LABPLAN; Naas, Ireland), dual indexed using Illumina sequencing adapters from the Illumina Nextera XT index kit v 2 (Illumina, San Diego, U.S.A.) and again purified using AMPure XP beads according to the Illumina 16S metagenomic sequencing library protocol. All libraries showed the expected size of ∼630 bp for V3–V4 region with an effective insert size of ∼460 bp flanked by adapters with a combined size of ∼170 bp after agarose gel electrophoresis. For all negative controls processed during the 16S library preparation, no bands were seen after agarose gel electrophoresis indicating the absence of cross-sample contamination.

Purifed barcoded amplicon libraries were quantified by a Qubit assay with the high sensitivity kit (Life Tehnologies, Carlsbad, CA, U.S.A.), normalized to 4 nM, and then combined in equal concentrations into a single pool according to same Illumina protocol. The library pool was diluted and denatured according to the Illumina MiSeq library preparation guide. The amplicon library (7 pM) was spiked with 20% denatured and diluted PhiX Illumina control v 3 (Illumina, San Diego, U.S.A.). The paired-end sequencing run was conducted on the Illumina MiSeq using the 600 cycle MiSeq reagent kit v 3 (Illumina, San Diego, U.S.A.).

### Bioinformatic and statistical analysis

Quantitative Insights Into Microbial Ecology (QIIME) pipelines were used to perform microbial community analysis. Dual-indexed sequence data were assembled by QIIME1 v 1.9.1 software then imported to QIIME2 v 2020.8 software package [17]. QIIME2 was then used to analyze the composition of the oral microbiota and determine the operational taxonomic units (OTUs), which corresponded to the 16S rRNA gene sequences in each sample.

After dereplication, sequences were clustered into individual OTUs at a default similarity level of 97% using closed reference picking strategy against reference 16S rRNA sequences (97_OTUs.fasta) from Greengenes database (gg 13_8_OTUs) [18]. Chimera identification was performed using uchime-denovo mode in QIIME2. After chimera filtering, Blast+ consensus taxonomy classifier was used to determine the taxonomic classification for each OTU using reference taxonomic reads from Greengenes database [18].

To detect the most significant taxa, the random forest classification algorithm was runned directly within QIIME2 via the QIIME sample-classifier tool. Random forest classification is a tree-based machine-learning algorithm based on the construction of multiple decision trees for making decisions [19]. A rooted phylogenetic tree was constructed using FastTree based on an OTU sequence alignment with MAFFT [20]. The α diversity analysis was performed to estimate both richness (number of taxonomic groups) and evenness (distribution of abundances of the groups) at species-level OTUs and genus-level OTUs with the nonparametric Shannon formula, using QIIME2 software [17].

The differences in diversity between all pairwise groups were assessed in QIIME2 using the Kruskal–Wallis test with a significance threshold for *P-value* of 0.05. α rarefaction curves were used to represent species richness measured by Shannon metric on rarefied samples to the lowest read depth of 4334 reads. To evaluate the relationship between oral microbial richness and age (as continuous variable), α diversity correlation was computed in QIIME2. To compare between coronary patients and controls, the Shannon index was measured with a *P*-*value* less than 0.05 considered as significant.

The β diversity was identified in QIIME2 using weighted (quantitative) and unweighted (qualitative) UniFrac phylogenetic distance metrics [21,22], using a random sample of 4334 sequences per sample. To compare β diversity between groups, the pairwise PERMANOVA (Permutational Multivariate Analysis of Variance) test was used with a *P-value* less than 0.05 considered as significant [23]. β diversity was also evaluated without phylogenetic consideration using Jaccard distance matrix on rarefied samples to 4334 reads.

To identify microbial signatures (specific taxa) associated with CAD, three methods for variable selection that acknowledge the compositional structure of microbiome data were used: analysis of composition of microbiomes (ANCOM) [24]; *selbal*, a forward selection approach for the identification of compositional balances [25]; and least absolute shrinkage and selection operator (LASSO), a penalized regression model for compositional data analysis [26]. In order to identify the most distinctive taxa between coronary patients and controls, we focus on the two methods *Selbal* and LASSO that share similar formulation as generalized linear models with specific constraints. The LASSO regression and *selbal* were applied to 65 genera obtained by filtering the relative frequencies based on their minimum frequencies higher that 1% and their presence in at least 20% of the total samples. For the selection of microbial balances with *Selbal*, the algorithm seeks for the two groups of taxa A and B whose relative abundances or balance B (A, B) is most associated with the outcome of interest Y according to the following generalized linear model:

$$\text{g}(\text{E}(\text{Y}))=\beta_{0}+\beta_{1}\times \text{B}(\text{A},\text{B})+{\text{g}}^{\prime }\text{Z}$$

where β_0_ is the intercept, β_1_ is the regression coefficient for the balance score, Z = (Z1, Z2,..., Zr) are additional non-compositional covariates, and γ is the vector of regression coefficients for Z. A cross-validation (CV) procedure was used to identify the set of balances. The mean squared error (MSE) as a function of the number of components included in the balance indicates the optimal number of variables identified in the forward selection process whose relative abundance is associated with the response variable of interest. *Selbal* algorithm is implemented as the R package *selbal* available on GitHub (https://github.com/UVic-omics/selbal). The second method, LASSO regression, is a regularized linear or logistic regression model allowing to select the most influential predictor/explanatory variables on a dependent variable by fitting a (linear or general linear) regression model with L1 penality. For an observed dependent variable y and a set of *k* explanatory variables measured on sample of *n* individuals *(y_i_, x_1i_,..., x_ki_), i = 1,..., n*, where in our case *y_i_* is a binary variable (such as status: coronary *vs* healthy) or a quantitative variable (such as Syntax score) and *x_i_ = (x_1i_,..., x_ki_)* are the composition of *k* taxa for sample *i*, LASSO fits a model of the form *y_i_ = β_0_ + β_1_ x_1i_ +···+ β_k_ x_ki_ + εi* and estimates the coefficients using least-squares method:

$$\sum_{i=l}^{n}\;{\left(y_{i}-\beta_{0}-1(x_{li})-\ldots -\beta_{k}(x_{ki})\right)}^{2}$$

under the constraint (which is an L1-penality)

$$\sum_{j\geq }|\beta_{j}|<t$$

which is equivalent to find the β coefficients that minimize the sum:

$$\sum_{i=l}^{n}\;{\left(y_{i}-\beta_{0}-\beta_{1}(x_{li})-\ldots -\beta_{k}(x_{ki})\right)}^{2}+\lambda \sum_{j\geq t}\left|\beta_{j}\right|$$

where λ is the penalization parameter that can be estimated using CV. LASSO shrinks some of the regression coefficients to zero, resulting in variable selection of the components with non-null coefficients. As suggested in a previous study [27], LASSO models were adjusted with both untransformed and Clr-transformed *x* variables. LASSO analyses were performed using the R package *glmnet* version (4.0.3).

To predict the functional composition from the 16S sequencing data, the OTUs were computed using PICTRUSt2 [28] and visualized by BURRITO software [29] to predict the functional pathways.

## Results

### Characteristics of study participants

A total of 20 CAD patients (age: 63.7±7.1 years, 19 females and one male), and ten controls (age: 52.6±8.3 years, ten females) not suffering from coronary syndromes were enrolled. All the participants were non-smokers. Clinical and laboratory data and the most documented medications are presented in Table 1.

**Table 1:** Characteristics of study participants

	CAD	Control	*P-value*
*n*	20	10	NA
Females, *n* (%)	19/20 (95%)	10/10 (100%)	NA
Age, years	63.4±7.1	52.6±8.3	0.0009
Body mass index (BMI)	26.8±3	28±3	0.35
Obesity (BMI > 30), *n* (%)	4/20 (20%)	2/8 (25%)	1
Strong adherence to the MD (4 ≤ score ≤ 8), *n* (%)	13/20 (65%)	5/10 (50%)	0.42
**Laboratory data**			
Known diabetes, *n* (%)	13/20 (65%)	3/8 (37.5%)	0.18
Known hypertension, *n* (%)	13/20 (65%)	3/8 (37.5%)	0.18
Hypercholesterolemia, *n* (%)	4/12 (33.33%)	0	NA
Dyslipidemia, *n* (%)	7/20 (35%)	2/8 (25%)	1
**Medications**			
Statin treatment, *n* (%)	17/20 (85%)	4/8 (50%)	0.053
β-blocker treatment, *n* (%)	7/19 (36.8%)	1/8 (12.5%)	0.36
Clopidogrel treatment, *n* (%)	9/20 (45%)	0	NA
**Clinical data**			
**Coronary artery stenosis**			
Non-significant stenosis (<50%), *n* (%)	11/20 (55%)	0	NA
Significant stenosis (≥50%), *n* (%)	9/20 (45%)	0	NA
Healthy, *n* (%)	0	10/10 (100%)	NA
**Syntax score classes**			
Low (≤16), *n* (%)	16/20 (80%)	0	NA
Intermediate (16–22), *n* (%)	2/20 (10%)	0	NA
High (>22), *n* (%)	2/20 (10%)	0	NA

NA: not applicable.

Compared with healthy controls, the BMI was not significantly different between CAD patient group and healthy subjects (*P=0.35*) and obesity was observed only for 25% of CAD patients and 20% of controls. Regarding nutritional habits, 65% of CAD patients were strongly adherent to the MD compared with 50% for controls (*P*=0.42). Common cardiovascular risk factors including diabetes, hypertension, hypercholesterolemia, and dyslipidemia were also compared between the two study groups and no significant differences were observed (Table 1). For clinical data, the coronary status of CAD patients was evaluated according to the coronary artery stenosis and the Syntax score. Of 20 patients, 11 (55%) presented non-significant stenosis and nine (45%) presented significant stenosis. Over CAD patients, 80% presented low Syntax score against 10% with intermediate and high Syntax score, respectively. All control individuals were free from CAD.

### The overall sequence data

We surveyed salivary bacterial communities of 20 patients with CAD and ten sex-matched controls. The V3–V4 regions of the bacterial 16S rRNA gene were PCR amplified. Following PCR amplification and agarose gel electrophoresis, no amplicon bands were observed in negative controls confirming the absence of contaminations. Purified, indexed, and pooled 16S metagenomic libraries were sequenced on the Illumina MiSeq system using v3 reagents. From the 30 samples, we generated a dataset of 3550054 high-quality 16S rRNA reads (minimum, 4334; maximum, 266295) with an average size of 465 nucelotide, which passed quality control. OTUs were created with the bioinformatics package QIIME2. Sequences were assigned to 731 OTUs using a 97% similarity threshold, and chimera checking revealed that 1.4% of total sequences were putative chimeras.

### Composition of the oral microbiota

After taxonomic assignment, the quality-filtered reads were clustered into 20 phyla, 30 classes, 55 orders, 100 families, 168 genera, and 208 species.

The identified OTUs were affiliated with 20 phyla that included 18 bacterial phyla, one bacterial candidate phylum, and one archaeal phylum Euryarchaeota.

**Figure 1:**
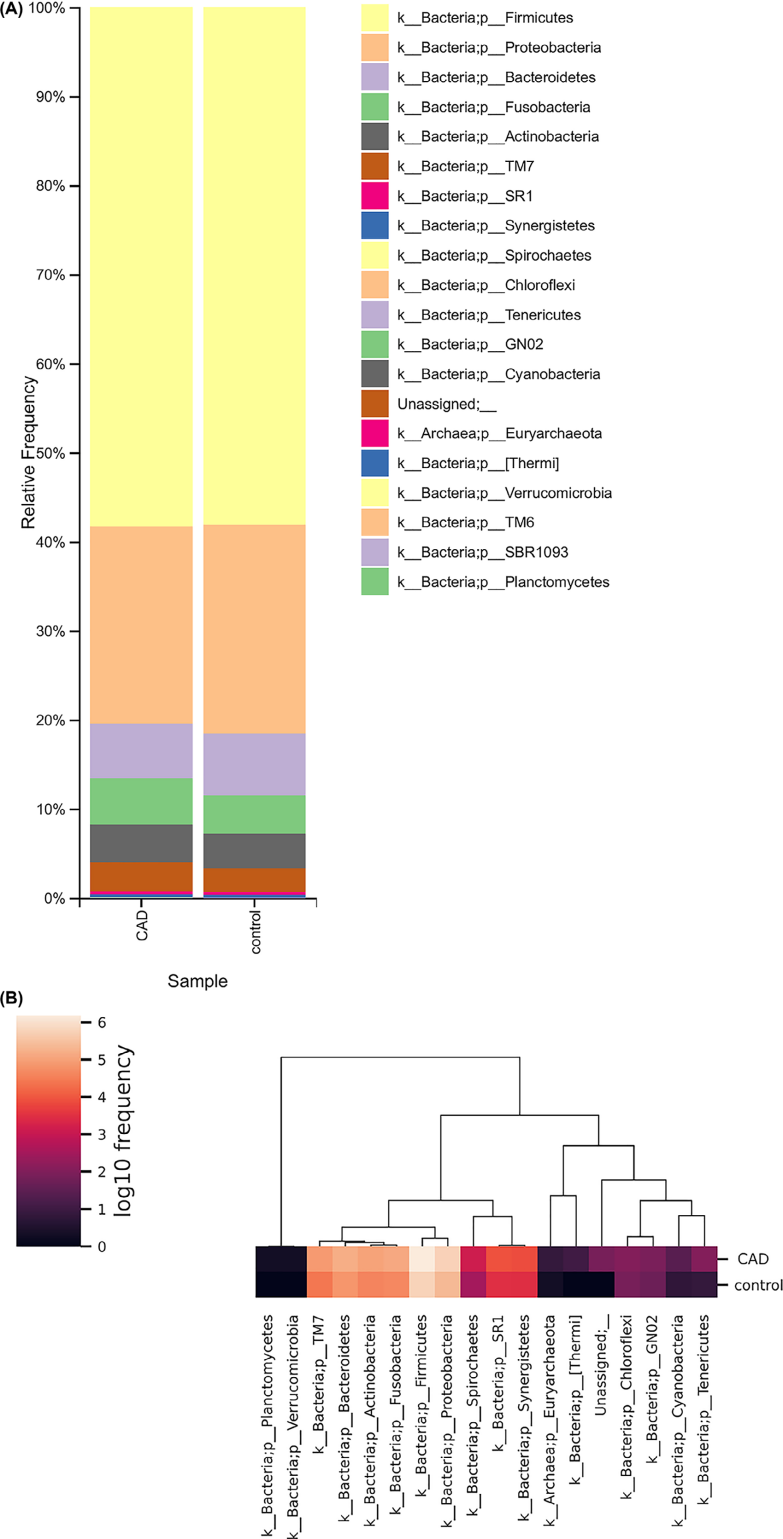
Salivary microbiome structure of coronary patients and controls on phylum level (**A**) The composition bar plots generated by QIIME2 pipeline showing the average relative abundance of each taxa. (**B**) Heatmap of 18 representative phyla selected by random forest classification algorithm. The heatmap illustrates the mean Log 10 frequency of each phyla in CAD patients and controls.

**Figure 2:**
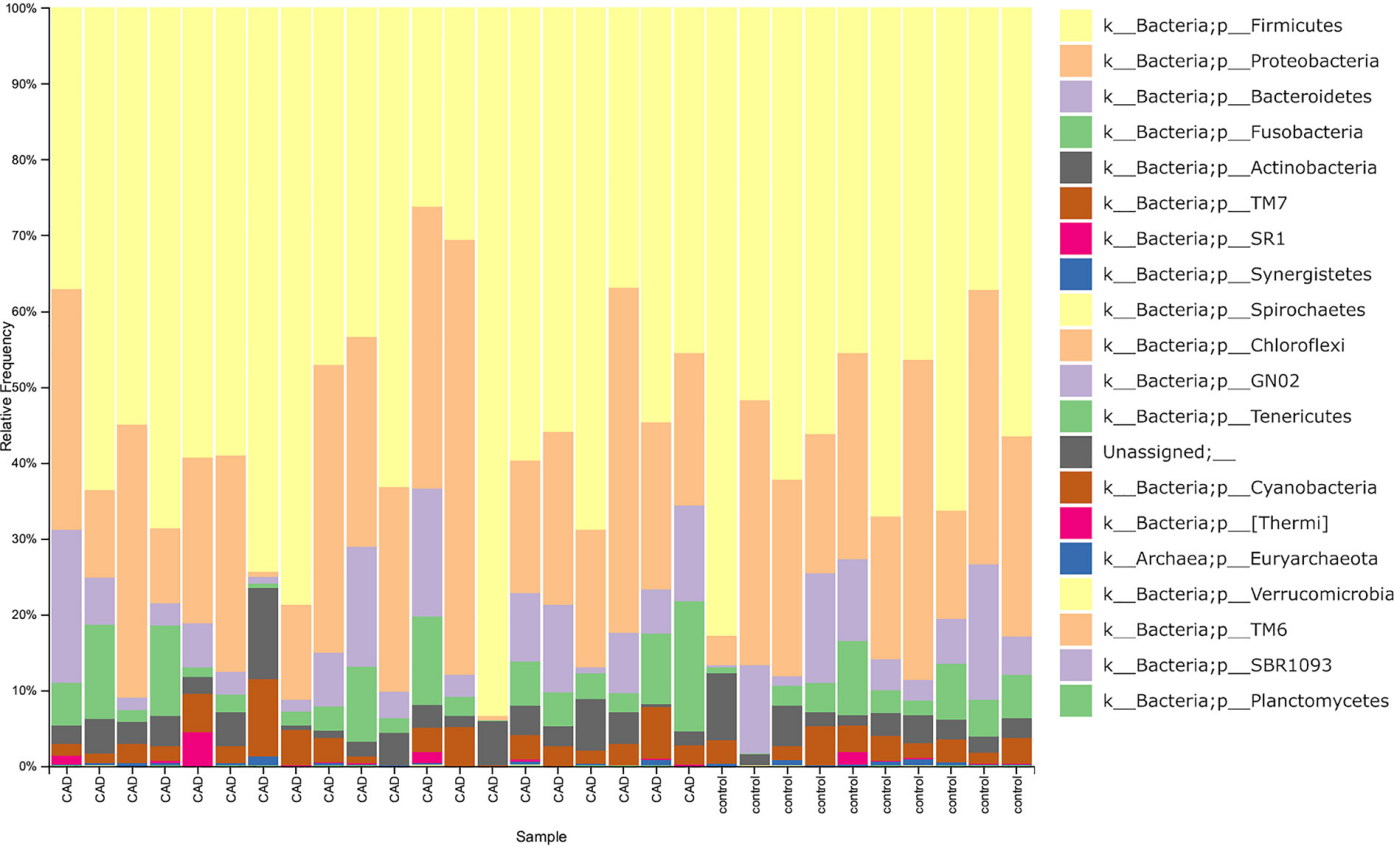
The composition bar plots for each sample on the phylum level

In the final dataset, 20/20 (100%) phyla were detected in CAD patients and 14/20 (70%) phyla were detected in controls (Figure 1A). The oral microbiota of patients and healthy controls was dominated by six bacterial phyla detected at relatively high abundance and classified as Firmicutes (58.33 and 58.14%), Proteobacteria (22.04 and 23.38%), Bacteroidetes (6.20 and 6.94%), Fusobacteria (5.15 and 4.31%), Actinobacteria (4.29 and 3.91%), and Saccharibacteria (TM7) (3.24 and 2.64%) (Figure 1A). The proportional ranges of these phyla were 26.26–93.49% for Firmicutes, 0.4–57.32% for Proteobacteria, 0.16–20.33% for Bacteroidetes, 0.01–17.15% for Fusobacteria, 0.53–12.08% for Actinobacteria, and 0–10.05% for Saccharibacteria (TM7). For rare bacterial taxa, the phyla Planctomycetes, SBR1093 and Verrucomicrobia were detected at the same time only in one CAD sample. The phylum TM6 was also detected in only one CAD sample. Moreover, the phyla [Thermi] and the unassigned bacterial candidate phyla were observed only in three and two CAD samples, respectively. The archaeal phylum Euryarchaeota was detected at 0.001% abundance average in two CAD samples and one control. The random forest classification algorithm selected 18/20 representative phyla using selected representative sequences (Figure 1B). The composition bar plots for each sample on the phylum level are shown in Figure 2.

On the genus level, a total of 168 different genera were detected including 162/168 (96.4%) and 122/168 (72.6%) in at least one sample from the CAD and the control groups respectively. Among these, 116/168 (69%) genera were present in both CAD and healthy groups.

The oral microbiota of patients and healthy controls was dominated by three genera including *Streptococcus* (24.97 and 26.33%), *Veillonella* (21.43 and 19.91%), and *Neisseria* (14.23 and 15.33%). Accordingly, these three most abundant genera accounted for 60.63 and 61.57% of the total taxa in the CAD and control groups, respectively. Moreover, eight genera were present at ≥1% relative abundance in all samples from the CAD and control groups including *Haemophilus* (5.17 and 5.48%), *Porphyromonas* (2.98 and 3.56%), *Rothia* (2.92 and 2.72%), *Fusobacterium* (2.84 and 1.92%), *Leptotrichia* (2.27 and 2.38%), *Granulicatella* (1.70 and 2.72%), *Prevotella* (2.16 and 1.85%), and *Selenomonas* (1.41 and 1.16%). In addition, only the archaeal genus *Methanobrevibacter* was detected in two CAD samples and one control with very low abundance relative frequency of 0.00129, 0.00236, and 0.000457%, respectively.

### Core-microbiome analysis

In the next step, we analyzed the OTUs at species level. A total of 208 different species were detected, including 200/208 (96%) species in CAD patients and 156/208 (75%) species in controls. Among these, 148/208 (68%) species were present in CAD and control individuals.

Using the most stringent definition of a 16S OTU being a member of a core microbiome in all samples (100%), we found a core ‘species-level’ phylotype represented by 22 species. Using a slightly less stringent threshold of 90%, we found 46 species including 14 assigned species (Table 2). Among these 46 species, ten species were detected at ≥1% abundance in CAD and control samples. The three most dominant species are *Streptococcus* sp. (23.68/25.38%), *Veillonella dispar* (20.91/19.46%), *Neisseria subflava* (12.81/13.31%); followed by less abundant species including: *Haemophilus parainfluenzae* (4.74/5%), *Porphyromonas* sp. (2.69/3.43%), *Rothia mucilaginosa* (2.57/2.43%), *Fusobacterium* sp. (2.84/1.92%), *Leptotrichia* sp. (2.27/2.38%), *Granulicatella* sp. (1.7/2.72%), and *Neisseria* sp. (1.4/2%). These species represent potential candidates for the core oral microbiome.

**Table 2:** Bacterial species detected in more than 90% of subjects

	Taxonomy			Prevalence n/N (%)	Abundance average % (CAD/controls)
	Phylum	Genus	Species		
100%	Firmicutes	*Veillonella*	*dispar*	30/30 (100%)	20.91%/19.46%
	Proteobacteria	*Neisseria*	*subflava*	30/30 (100%)	12.81%/13.31%
	Proteobacteria	*Haemophilus*	*parainfluenzae*	30/30 (100%)	4.74%/5%
	Actinobacteria	*Rothia*	*mucilaginosa*	30/30 (100%)	2.57%/2.53%
	Bacteroidetes	*Prevotella*	*melaninogenica*	30/30 (100%)	1.19%/0.97%
	Firmicutes	*Bulleidia*	*moorei*	30/30 (100%)	0.49%/0.44%
	Firmicutes	*Streptococcus*	*anginosus*	30/30 (100%)	0.64%/0.64%
	Firmicutes	*Selenomonas*	*noxia*	30/30 (100%)	0.26%/0.17%
	Actinobacteria	*Rothia*	*dentocariosa*	30/30 (100%)	0.31%/0.13%
	Bacteroidetes	*Porphyromonas*	*endodontalis*	30/30 (100%)	0.29%/0.13%
	Firmicutes	*Streptococcus*	sp.	30/30 (100%)	23.68%/25.38%
	Firmicutes	*Granulicatella*	sp.	30/30 (100%)	2.38%/2.72%
	Bacteroidetes	*Porphyromonas*	sp.	30/30 (100%)	2.69%/3.43%
	Fusobacteria	*Leptotrichia*	sp.	30/30 (100%)	2.27%/2.38%
	Proteobacteria	*Neisseria*	sp.	30/30 (100%)	1.4%/2%
	Fusobacteria	*Fusobacterium*	sp.	30/30 (100%)	2.84%/1.92%
	Firmicutes	*Selenomonas*	sp.	30/30 (100%)	1.13%/0.98%
	Bacteroidetes	*Capnocytophaga*	sp.	30/30 100%	0.8%/0.95%
	Actinobacteria	*Atopobium*	sp.	30/30 (100%)	0.37%/0.43%
	Actinobacteria	*Actinomyces*	sp.	30/30 (100%)	0.63%/0.49%
	Proteobacteria	*Aggregatibacter*	sp.	30/30 (100%)	0.54%/0.7%
	Bacteroidetes	*Prevotella*	sp.	30/30 (100%)	0.35%/0.24%
>90%	Proteobacteria	*Aggregatibacter*	*segnis*	28/30 (93,3%)	0.18%/0.1%
	Actinobacteria	*Corynebacterium*	*durum*	28/30 (93,3%)	0,04%/0.12%
	Bacteroidetes	*Prevotella*	*nanceiensis*	28/30 (93,3%)	0.08%/0.14%
	Bacteroidetes	*Capnocytophaga*	*ochracea*	27/30 (90%)	0.6%/0.98%
	Firmicutes	*Dialister*	sp.	29/30 (96,66%)	0.27%/0.27%
	Firmicutes	*Streptococcus*	sp.	29/30 (96,66%)	0.41%/0.21%
	Proteobacteria	*Eikenella*	sp.	28/30 (93,3%)	0.05%/0.06%
	Firmicutes	*Oribacterium*	sp.	28/30 (93,3%)	0.83%/0.97%
	Firmicutes	*Veillonella*	sp.	28/30 (93,3%)	0.35%/0.3%
	Proteobacteria	*Haemophilus*	sp.	28/30 (93,3%)	0.44%/0.48%
	Bacteroidetes	*Tannerella*	sp.	28/30 (93,3%)	0.08%/0.09%
	Firmicutes	*Moryella*	sp.	28/30 (93,3%)	0.12%/0.04%
	Firmicutes	*Schwartzia*	sp.	28/30 (93,3%)	0.04%/0.12%
	Proteobacteria	*Cardiobacterium*	sp.	28/30 (93,3%)	0.08%/0.14%
	Proteobacteria	*Kingella*	sp.	28/30 (93,3%)	0.03%/0.06%
	Proteobacteria	*Campylobacter*	sp.	28/30 (93,3%)	0.95%/0.7%
	Bacteroidetes	*Paludibacter*	sp.	28/30 (93,3%)	0.03%/0.04%
	Bacteroidetes	*Prevotella*	sp.	28/30 (93,3%)	0.08%/0.06%
	Firmicutes	*Veillonella*	sp.	28/30 (93,3%)	0.06%/0.07%
	Firmicutes	*Parvimonas*	sp.	27/30 (90%)	0.62% /0.84%
	Synergistetes	*TG5*	sp.	27/30 (90%)	0.29%/0.29%
	Firmicutes	*Filifactor*	sp.	27/30 (90%)	0.27%/0.33%
	Firmicutes	*Megasphaera*	sp.	27/30 (90%)	0.39%/0.45%
	Firmicutes	*Butyrivibrio*	sp.	27/30 (90%)	0.05%/0.04%

### Comparison of oral microbiota between control and cases

**Figure 3:**
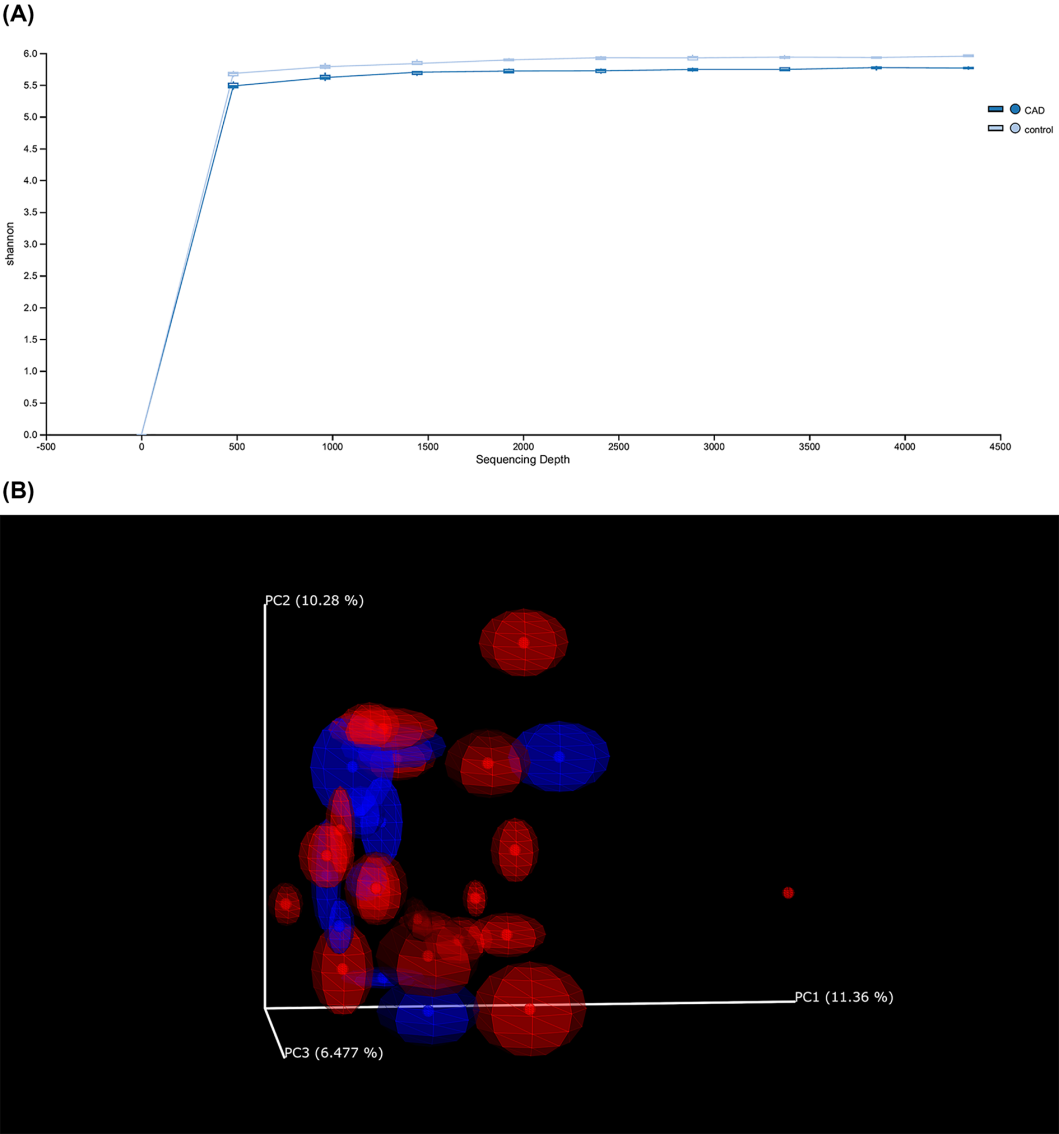
α and β diversity rarefaction (**A**) α rarefaction curves showing species richness using Shannon metric on rarefied samples to the lowest read depth of 4334 reads. (**B**) Principal Coordinate Analysis (PCoA) plot of β diversity rarefaction using Jaccard distances calculated between samples rarefied to the lowest read depth of 4334 reads showing an outlier CAD sample.

At the species level, statistical analysis showed that the oral microbiome richness and evenness of the CAD group were not significantly different compared with the control group with *P-value* of 0.17 (Shannon diversity index) and 0.15 (Shannon evenness index). However, at the same taxa scale, a significant slightly higher richness was observed in healthy group compared with CAD patients with significant stenosis (*P=*0.041). Furthermore, analyses did not reveal a significant difference in α diversity at the genus-level OTUs between the healthy and patient oral samples (*P-values* of 0.86 for Shannon diversity index and 0.86 for Shannon evenness index). α rarefaction curves showed saturated plateaus in all samples within both groups and the control samples plotted in the upper part of the graph (Figure 3A). The difference in α diversity between pairwise groups was also evaluated using the Kruskal–Wallis test to assess associations between other clinical parameters and microbial profiles. At species-level OTUs, with regard to statin treatment, a significant difference in evenness was observed between treated and not-treated individuals (*P*=0.027). At the genus-level OTUs, a significant difference in richness and evenness was observed between CAD patients with low Syntax score compared with those with moderate Syntax score (*P*=0.035; *P*=0.035). Regarding adherence to the MD, no significant difference in α diversity between strong and poor adherents was found (*P*=0.09 for Shannon diversity index; *P*=0.77 for evenness). For age as a continuous variable, we observed no correlation between microbial richness and age for both groups (*P-values* of 0.51 for Shannon index).

Regarding β diversity, the phylogeny-based assessment of differences in overall bacterial community composition was performed using the UniFrac distance metric. Analysis revealed no significant differences in diversity between the CAD and control groups with *P* of 0.77 (weighted-UniFrac) and 0.48 (unweighted-Unifrac). PCoA plot using Jaccard distance matrix of β diversity rarefaction showed an outlier CAD sample (Figure 3B). β diversity analysis did not reveal significant difference that could discriminate between other pairwise groups.

### Variable selection in microbiome compositional data

The relative abundances of 65 genera, filtered at ≥1% abundance in at least 20% of samples, were compared between the CAD and control groups by means of *Selbal* and LASSO penalized regression models.

**Figure 4:**
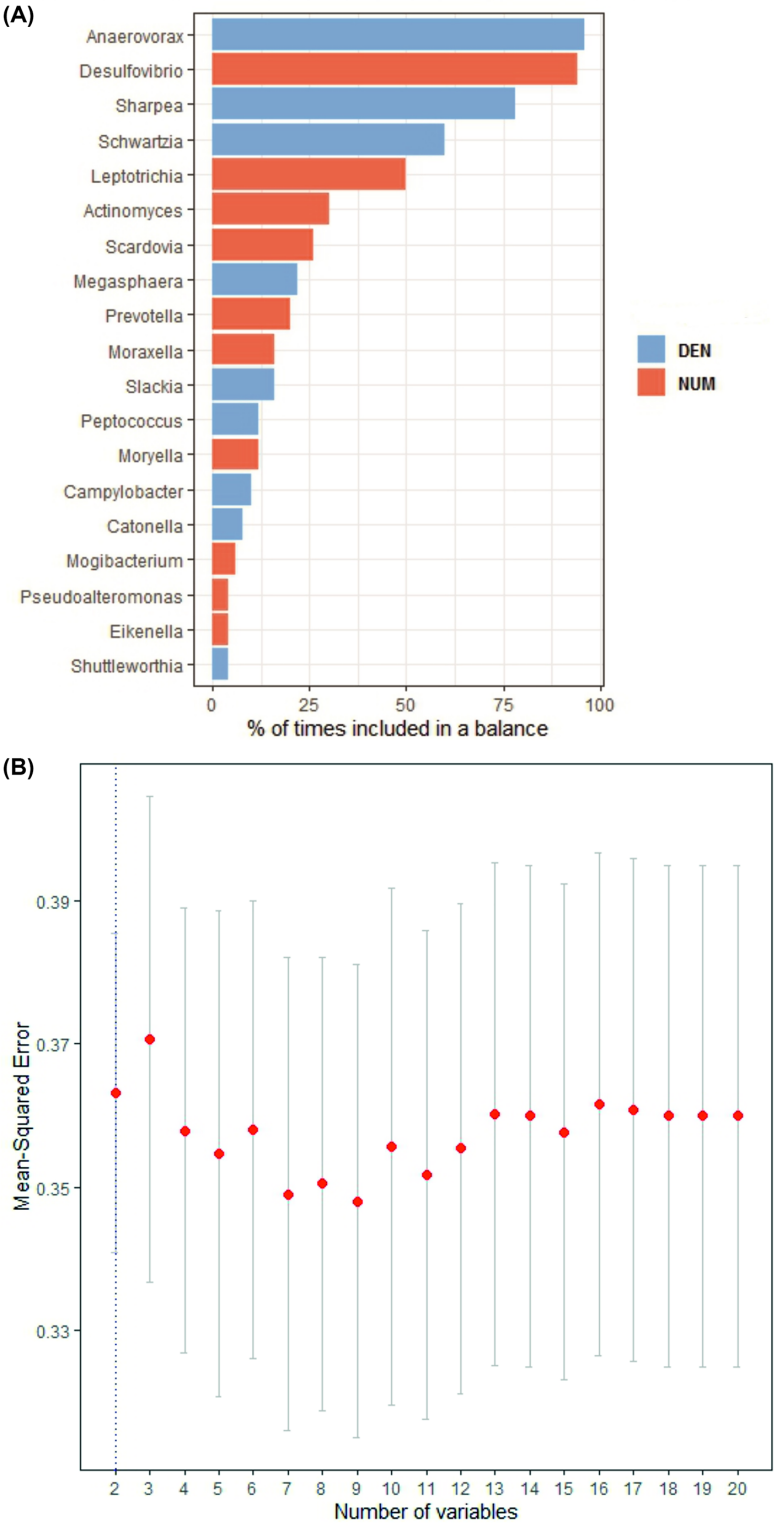
Description of the global balance for CAD *by Selbal* methodology (**A**) CV results for CAD study showing the most frequent taxa selected in the CV procedure compared with the global balance obtained with the whole data set. Colored rectangles indicate if the component is in the numerator (NUM) (red) or in the denominator (DEN) (blue) of the balance. (**B**) MSE as a function of 19 components included in the balance.

The goal of *selbal* methodology is to identify microbial signatures that are able to discriminate between CAD and non-CAD individuals. According to the *Selbal* method, the two groups of taxa defining the global balance are group A = [*Desulfovibrio*, *Leptotrichia, Actinomyces*, *Scardovia*, *Prevotella*, *Moraxella, Moryella*, *Mogibacterium*, *Pseudoalteromonas*, and *Eikenella*] and group B = [*Anaerovorax*, *Sharpea, Schwartzia*, *Megasphaera*, *Slackia*, *Peptococcus*, *Campylobacter*, *Catonella*, and *Shuttleworthia*] (Figure 4A). The average abundance (geometric mean) of taxa in group B relative to group A is approximately the same in controls than in coronary patients. The number of taxa that yields the MSE is 19 (Figure 4B). However, a similar level of MSE is provided by two microbial taxa, *Desulfovibrio* and *Anaerovorax*, representing 70% of balances in the dataset between CAD and Control.

**Figure 5:**
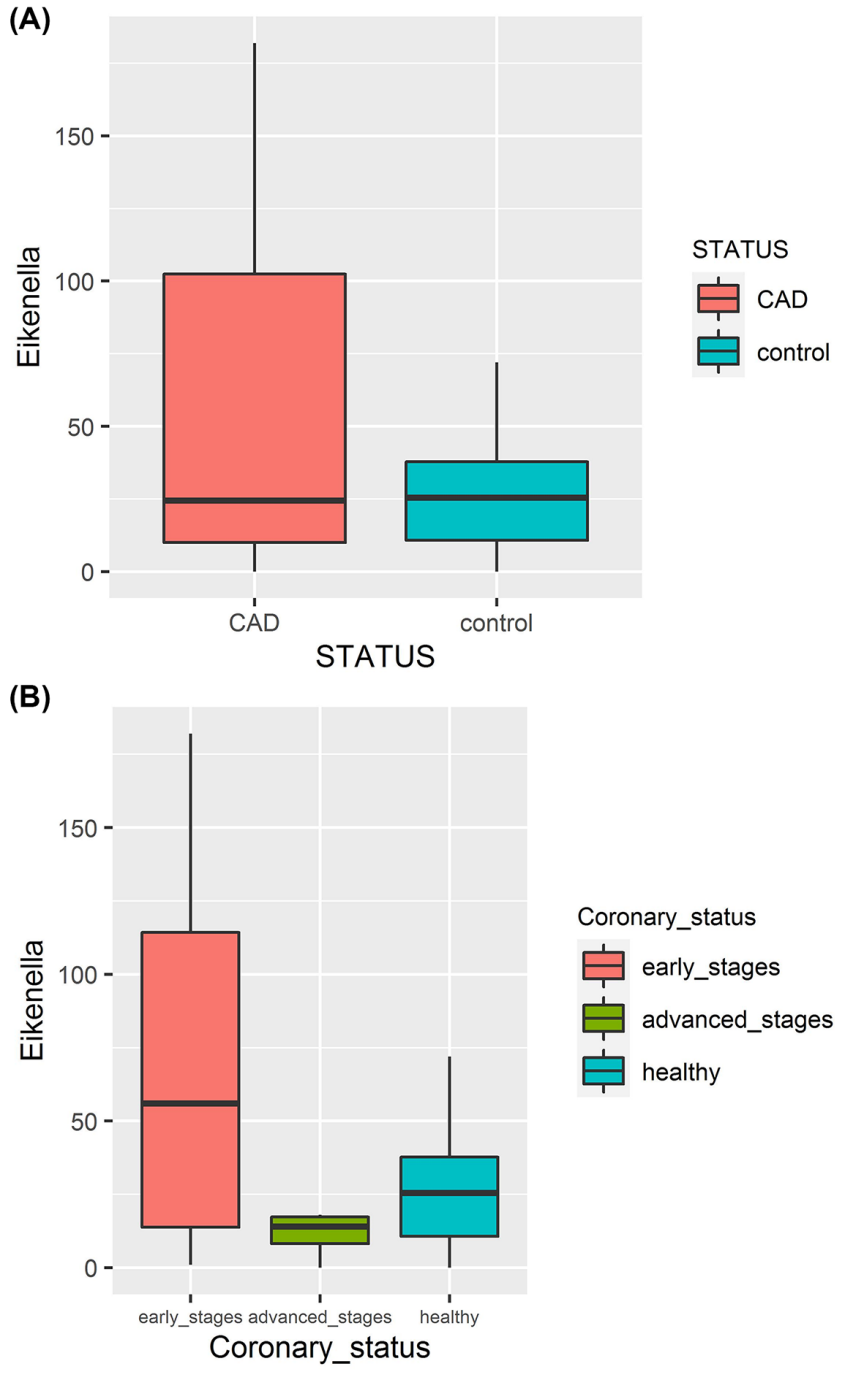
Box plots showing the absolute frequency of the bacterial genus *Eikenella* (**A**) Abundance of the bacterial genus *Eikenella* in CAD patients compared with the control group. (**B**) Abundance of *Eikenella* in CAD patients at early and advanced stages, and the control group. The *y* axis represents the absolute frequency of *Eikenella*.

Model selection using LASSO penalized regression (binary logistic regression for status and linear regression for Syntax score, respectively) was carried on to identify the most discriminant OTUs between controls and patients or most associated with syntax score within patients. The Syntax score was recoded as a binary variable: early stages (<10) (*n*=13) and advanced stages (≥10) (*n*=7) to balance size classes. λ values of LASSO were 0.167 and 5.421 for the two models, respectively. The results identified a single OTU *Eikenella* as the most influential for both status and syntax score. Figure 5 shows a higher abundance of the bacterial genus *Eikenella* in CAD patients particularly at early stages compared with the control group (Figure 5). Log-transform or clr-transform of the OTU abundance did not yield any change in the results. Bivariate analyses correlating status (diseased *vs* controls) and Syntax score (in CAD patients) using appropriate tests (Student/Mann–Whitney tests and Spearman correlation coefficient, respectively) showed, after FDR correction for multiple testing, no significant association of disease status with any of the OTUs but a significant negative correlation of Syntax score with OTU *Eikenella* (Spearman rho = *−0.68*, *P*=*0.00094*), which is more abundant in early-stage patients with lower syntax score.

However, the ANCOM results, which were based on bacterial genera, showed no differences between the CAD and control groups (W = 386).

### Comparison of functional pathways in the saliva microbiota between CAD patients and controls

The functional composition from the 16S sequencing data of cases and controls was computed using PICTRUSt2. A total of 391 predictive functional pathways were observed in both groups (Supplementary Table S1). BURRITO software classified the functional pathways on five categories including cellular processing, environmental information processing, genetic information processing, metabolism, and unclassified. Visualization by BURRITO software showed that functional pathways were dominated by the metabolic pathways same in cases and controls represented by more than 25% of the average relative abundance of function. In addition, the metabolic pathways were contributed by the most abundant OTUs represented by Firmicutes and Proteobacteria with an average taxon-function attribution >25%.

## Discussion

The present study describes the oral microbiota composition of a patient group suffering from CAD and a healthy control group for the first time in North Africa. The present study also provides the inter-individual comparison between both groups necessary to elucidate whether the oral microbiota composition was altered in diseased patients.

Salivary microbiome profiling was performed by high-throughput 16S rRNA sequencing using the Illumina Miseq platform. This approach allowed accurate characterization of the complex oral microbiome with high sensitivity and rapidity; providing information of microbial diversity including uncultivable microorganisms and facilitating phylogeny and taxonomy analysis. This strategy was also adopted in the previous studies to characterize the oral microbial communities in CAD patients compared with healthy controls [11,12,14].

We found that oral microbial communities in patients and healthy controls are represented by similar global core oral microbiome. The most common phyla for patients and controls were Actinobacteria (A), Bacteroidetes (B), Firmicutes (F), Fusobacteria (Fu), Proteobacteria (P), and Saccharibacteria (TM7) (T). This observation is in accordance with the current knowledge of oral composition with differences in phyla relative abundance [30,31].

Compared with the previous studies, different abundance rank order were observed: F > P > B > Fu > A > T for our study, F > B > A > Fu > P [11] and F > A > B > P > Fu [12] for Swedish studies, and F > P > Fu > B > A > T for Indian [14]. While DNA was extracted from saliva in our study, oral swabs were sampled in the other publications [11,12,14]. The sampling site and the geographical origin of each study population may affect the detected phyla and their relative abundance. Differences in phyla diversity were not significant between diseased and healthy groups in all studies.

At the genus level, the predominant taxa with comparable abundance for both groups belonged to *Streptococcus*, *Veillonella*, *Granulicatella, Selenomonas* (phylum Firmicutes); *Neisseria*, *Haemophilus* (phylum Proteobacteria); *Rothia* (phylum Actinobacteria); *Prevotella*, *Porphyromonas* (phylum Bacteroidetes); and *Fusobacterium, Leptotrichia* (phylum Fusobacteria).

The genera *Streptococcus* and *Veillonella* were the most dominant taxa in all individuals with more than 20% relative abundance in CAD patients and in controls. For these genera, no significant difference was found between both groups. These bacterial genera were also reported with the highest relative abundances in the oral microbiota of coronary patients and controls in the previous studies [12,13]. *Streptococcus* and *Veillonella* are early colonizers during the process of biofilm formation in the oral cavity initiated by the aggregation of these bacteria to establish an initial microenvironment [8]. Interestingly, these two genera were also detected in atheromatous plaques and were considered as plaque-associated bacteria. These migratory bacteria can form biofilm structures within atherosclerotic plaques when entering into the coronary vasculature [8].

To identify a microbial signature defined by two groups of taxa whose relative abundances, or balance, are associated with CAD status, *selbal* analysis was performed. The microbial signature that best discriminates between CAD and controls was represented by the balance between taxa in group *A* [*Desulfovibrio*, *Leptotrichia, Actinomyces*, *Scardovia*, *Prevotella*, *Moraxella, Moryella*, *Mogibacterium*, *Pseudoalteromonas*, and *Eikenella*] and group *B* [*Anaerovorax*, *Sharpea, Schwartzia*, *Megasphaera*, *Slackia*, *Peptococcus*, *Campylobacter*, *Catonella*, and *Shuttleworthia*]. *Anaerovorax* and *Desulfovibrio* were the most frequent taxa included in about 70% of CV balances. Except *Anaerovorax, moraxella*, and *pseudoalteromonas*, the remaining 16 taxa selected by *Selbal* in both groups were associated with dental caries or periodontal diseases as gingivitis and periodontitis [32–39]. Previously, the periodontal pathogen burden has been linked with coronary heart disease [32,40]. Periodontal bacteria have also been found in atherosclerotic lesions [8]. Furthermore, several epidemiological studies and meta-analyses established a robust association between periodontal disease and increased risk for coronary heart disease confering approximately a 24–35% increase in risk of CAD [41,42]. As periodontal diseases are associated with dysbiosis of the oral microbiota, it was suggested that the subgingival microbiota plays a role in systemic inflammation and atherogenesis [32]. This fact constitutes an active lever for systemic subclinical inflammation enhancement and eventually contributes to endothelial and vascular dysfunction.

Using the penalized regression analysis LASSO, the genus *Eikenella* was the major discriminant taxon between coronary patients and the control group. The significant negative correlation of syntax score with *Eikenella* observed in coronary patients group could suggest the implication of this genus mainly in the initial pathogenesis phase of atherosclerosis. Accordingly, the highest abundance of *Eikenella* observed in oral coronary patient samples particularly at early stages could potentially be a prominent pathological indicator for the development of atherosclerosis. To our knowledge, we report for the first time an association between CAD and an increased abundance of *Eikenella* in the oral cavity.

In the previous studies, while abundance of *Anaeroglobus* in the oral cavity was associated with symptomatic atherosclerosis [12], CAD was not related to significant qualitative changes of the oral microbiota compared with healthy controls in other studies [11,13,14].

*Eikenella* is a Protobacteria bacterium usually found in the oral cavity and gastrointestinal tract reported as a periodontal pathogen [38,43]. This periodontitis-related bacterium induces oral epithelial cells to express inflammatory mediators (interleukin-6 (IL-6), IL-8, tumor necrosis factor-α (TNF-α), prostaglandin E2, and cyclooxygenase-2) without requiring direct contact with these cells [44]. Therefore, it was suggested that soluble components from *Eikenella* *corrodens* species may induce the expression of various inflammatory mediators [45]. In addition, the species *E. corrodens* is a member of fastidious microorganisms group referred as HACEK (*Haemophilus* species, *Aggregatibacter* species, *Cardiobacterium hominis*, *E. corrodens*, and *Kingella* species) recognized as a cause of infective endocarditis associated with a higher prevalence of immunologic/vascular manifestations and stroke [46]. Accordingly, *E. corrodens* implication in infective endocarditis has been reported since 1983 [43] and its presence in atherosclerotic plaques was reported in several studies raising the possibility that it may directly affect the pathogenesis of atherosclerosis [8]. Recently, the proinflammatory effects of *E. corrodens* lipopolysaccharide (LPS) on human coronary artery endothelial cells (HCAECs) were investigated [47]. The present study indicated that the periodontopathic endotoxin *E. corrodens*-LPS induced proinflammatory response in HCAECs through TLR4, ERK, and NF-jB p65, triggering a pro-atherosclerotic endothelial response and enhancing monocyte adhesion [47]. Therefore, the periodontal pathogen *E. corrodens* could be a major contributor enhancing the atherosclerotic process by inducing endothelial inflammation [47].

Although the exact mechanism remains to be identified, our analysis revealed that the genus *Eikenella* is a key bacterial member of the oral microbiota in CAD patients that may affect the atherogenesis. Further studies with a larger sample number from different Northern Africa populations are needed to convincingly conclude that *Eikenella* could be used as a pathological marker for early CAD diagnosis.

In summary, we detected similar core bacterial members of the oral microbiota in patients and healthy controls and a different abundance distribution of the genus *Eikenella* in coronary patients compared with controls. The oral microbiota was characterized for the first time in diseased and healthy individuals in North Africa and the differential relative abundance of *Eikenella* was reported for the first time compared with equivalent studies. Limitations of the present study include the number of patients investigated that may have underpowered to detect additional subtle changes in the oral microbiota between CAD patients and controls.

Our findings reporting the higher abundance of *Eikenella* in oral coronary patient samples compared with controls should be evaluated in further studies to better explore this potential oral microbiome signature associated with coronary disease.

## Supplementary Material

Supplementary Table S1

## Data Availability

Sequence data from the present article have been deposited with GenBank Data Libraries under Accession No. MW886334–MW887523.

## Abbreviations

ANCOM: analysis of composition of microbiomes
BMI: body mass index
CAD: coronary artery disease
CVD: cardiovascular diseases
CV: cross-validation
DEN: denominator
HCAEC: human coronary artery endothelial cell
IL-6: interleukin-6
LASSO: least absolute shrinkage and selection operator
LPS: lipopolysaccharide
MSE: mean squared error
MD: mediterranean diet
NA: not applicable
NUM: numerator
OTU: operational taxonomic unit
PCoA: principal coordinate analysis
PERMANOVA: permutational multivariate analysis of variance
QIIME: quantitative insights into microbial ecology
rRNA: ribosomal RNA
TNF-α: tumor necrosis factor-α
